# Five new species of *Rhodamnia* (Myrtaceae, Myrteae) from New Guinea

**DOI:** 10.3897/phytokeys.19.4098

**Published:** 2012-12-28

**Authors:** Neil Snow

**Affiliations:** 1 Herbarium Pacificum, Bishop Museum, 1525 Bernice St., Honolulu, HI 96821 USA; 2 527 S. Oakes St, Helena, MT 59601 USA

**Keywords:** Australia, conservation, Myrtaceae, New Guinea, new species, *Rhodamnia*, systematics

## Abstract

Five new species of *Rhodamnia* are proposed for New Guinea, including *Rhodamnia asekiensis*, *Rhodamnia daymanensis*, *Rhodamnia makumak*, *Rhodamnia taratot*, and *Rhodamnia waigeoensis*. *Rhodamnia sharpeana*, known previously only in Australia, is reported for the first time for Papua New Guinea. Detailed species descriptions and associated taxonomic data are provided for all species. A key is provided for species of *Rhodamnia* with stellate trichomes. Given the overall paucity of collections, all species are tentatively assigned as Data Deficient following IUCN conservation recommendations.

## Introduction

*Rhodamnia* Jack is recognized easily among the baccate genera of Myrtaceae in Malesia and Melanesia by its 4-merous flowers (apart from a 5-merous species in New Caledonia), uniloclular ovaries, parietal placentation, and sclerotic seed coats (Scott 1979; Snow 2007).

Based on the author’s current taxonomic perpectives and including those newly proposed here, *Rhodamnia* includes 42 species. The genus occurs from Myanmar, Thailand and China through Malesia and Australia, and east through Melanesia to the Solomon Islands (Scott 1979; Kress et al. 2003). The Australian species recently were treated (Snow 2007) and another new species was described from New Guinea (Snow and Takeuchi 2009). However, *Rhodamnia* remains imperfectly known in New Guinea because of low collecting densities from that island, and because some species are known from only one or a few collections.

The five species newly proposed here were recognized during curatorial duties associated with floristic inventories in Papua New Guinea by Bishop Museum. The purpose of this paper is to describe the new species of *Rhodamnia*, summarize their diagnostic character traits, and to provide a distribution map and conservation threat assessments. It also discusses the first New Guinean occurrences of *Rhodamnia sharpeana*, previously known only from Australia (Snow 2007).

## Materials and methods

Measurements are based primarily on dried herbarium specimens, although dimensions for flowers and fruits were supplemented by rehydrating material in boiling water. Terminology follows that used in recent treatments for the genus (Snow 2007; Snow and Takeuchi 2009), the Systematics Association (1962) for two-dimensional shapes, and Thiers (2012) for acronyms. An exception is the present use of the term colleters in lieu of stipules in light of recent studies (da Silva et al. 2012). Descriptions for color are standardized where possible to Beentje (2010) or reported in accordance with data provided on specimen labels. As used here, the leaf apex refers to the distal 25% of the laminar surface, whereas the tip refers to the distal 10% (Snow et al. 2003). Conservation threat assessments follow IUCN (2010). Takeuchi (2000) discussed the inconsistency of vernacular names in Papua New Guinea and provided an example from Myristicaceae in which a common name was applied widely across most of the family. "However, the vernacular name is reported here if it was indicated on the specimen label.

### Data resources

The data underpinning the new species described in this paper are deposited at GBIF, the Global Biodiversity Information Facility, http://ipt.pensoft.net/ipt/resource.do?r=snow_1_rhodamnia_new_guinea.

### Rhodamnia asekiensis

N. Snow sp. nov.

urn:lsid:ipni.org:names:77123882-1

http://species-id.net/wiki/Rhodamnia_asekiensis

Figures 1 2

*Resembling* Rhodamnia latifolia *but with distinctly larger leaves that have an acute to acuminate apex and differing by the larger fruits*.

#### Type.

Papua New Guinea. Morobe Province, Aseki, Menyama Subdistrict, 7°21'S, 146°10'E, 20 May 1968, H. Streimann & A. Kairo NGF 39049(holotype: BISH [sheet no. 30914]!; isotypes: A!, CANB!, K!, L!, LAE n.v., NSW n.v., US!)

#### Description.

Trees of unknown height; crown dense. Bark of main bole light grey, vertically fissured. Indumentum (branchlets, flowers, fruit) short-sericeous, sparsely to moderately dense, color more or less saffron (Beentje 2010). Branchlets terete, wingless, dark brown (dried); epidermis smooth, oil glands absent. Leaves opposite, evenly distributed along branchlets, strongly discolorous; venation perfect basal or slightly suprabasal acrodromous, secondary and tertiary veins visible above and below, the more prominent secondaries ca. 20–25 per side abaxially, the secondaries near base of blade splitting as they approach lateral primary vein and contrasting with those towards apex of blade that are mostly unbranched; intramarginal vein less pronounced than secondaries, parallel to leaf margins, 0.8–1.1 mm from margin at midpoint of blade. Colleters absent. Petioles 11–13 mm long, round to slightly sulcate above. Leaf blades 10.5–16.0 cm long, 3.5–4.5 cm wide, narrowly ovate (to elliptic), base cuneate, apex acuminate, tip acute; adaxial surface matte, glabrescent at base, midvein slightly and narrowly raised proximally but becoming flush distally; abaxial surface densely short and strongly-appressed sericeous between the secondary and tertiary veins, midvein projecting throughout, oil glands not visible. Inflorescence terminal or lateral, flowers solitary (=monads) or in 3-flowered cymes (“botryoids” of some authors), pedicels of monads up to 10 mm long. Bracteoles not seen, apparently caducous in fruit. Flowers unknown. Hypanthium (based on fruit) evidently not ribbed, hairy. Calyx lobes (in mature fruit) 2.5–3.0 mm long, more or less glabrous adaxially, moderately sericeous abaxially, persisent and erect in fruit. Ovary (from mature fruit) and locule 1, placentas 2, linear; ovules disposed in regular rows. Berries subcylindrical, somewhat pyriform or tapering at the base, 8.5–14.0 mm long, 8–11 mm wide, sparsely sericeous, dull dark red (fresh) or blackish (dried). Seeds 4–10 per fruit, 4.5–5.2 mm long, 2.8–5.0 mm wide, rounded on outer portion adjacent to fruit wall but highly angular and irregularly elsewhere, light brown, seed coat highly sclerotized. Embryos not seen.

**Figure 1.**
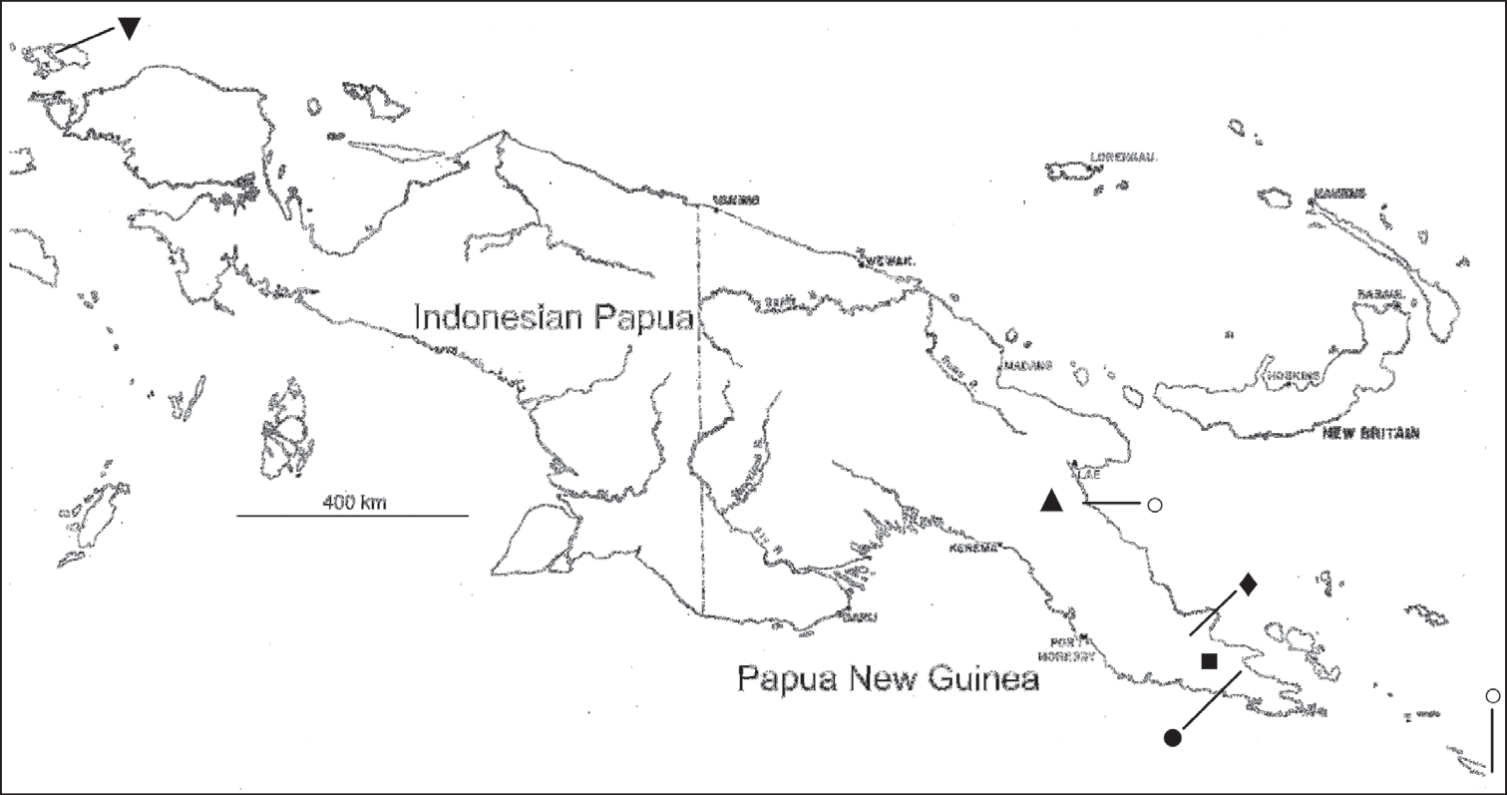
Island of New Guinea, showing localities of new species. Triangle (▲) = *Rhodamnia asekiensis*; Square (■) = *Rhodamnia daymanensis*; Diamond (◆) = *Rhodamnia makumak* (at bottom of line); Closed circle (⚫) = *Rhodamnia toratot* (at top of line); Inverted triangle (▼) = *Rhodamnia waigeoensis* (upper left, at bottom of line); Open circles (⚪) = *Rhodamnia sharpeana* (left end of line [upper] and bottom of line [lower, on truncated eastern half of Tagula Island]).

**Figure 2.**
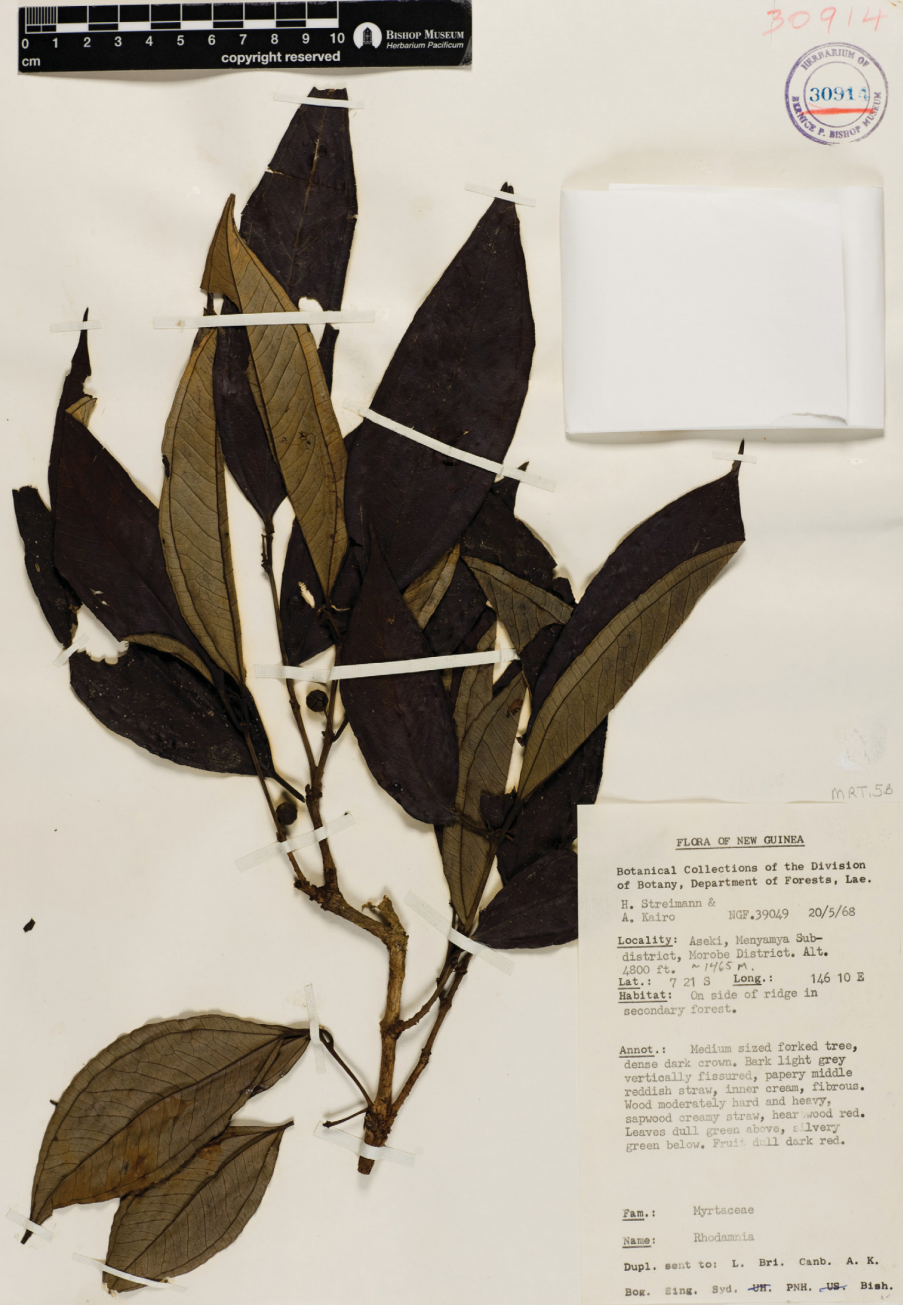
*Rhodamnia asekiensis* N. Snow. Photo of the holotype at BISH (*H. Streimann and A. Kairo NGF 39049*).

#### Phenology.

Flowering unknown; fruiting confirmed only for May.

#### Distribution.

Known only from Morobe Province, Papua New Guinea, on the side of a ridge in a secondary forest; ca. 1465 m.

#### Conservation status.

Data Deficient; but the subsequent lack of collections of this species over the past 45 years locally and regionally suggest that Threatened might more accurately reflect is true status.

#### Comments.

*Rhodamnia asekiensis* is included among the “pearly” group of species (Snow 2007) by virtue of its nacreous abaxial laminar indumentum. The 10–16 cm long leaves with the acute to acuminate apices distinguish it from *Rhodamnia latifolia*, in which Scott (1979) earlier had placed the type specimen.

### Rhodamnia daymanensis

N. Snow sp. nov.

urn:lsid:ipni.org:names:77123883-1

http://species-id.net/wiki/Rhodamnia_daymanensis

Figures 1 3

*Resembling* Rhodamnia lancifolia *but differing by its more deeply sulcate petiole, broader leaves, and shorter yellowish indumentum on the abaxial laminar surface*.

#### Type.

PapUa New Guinea. Milne Bay District, north slopes of Mt. Dayman, Maneau Range, 2250 m, ca. 9°47'S, 149°18'E, 2 Jun 1953, L. J. Brass 22718 (holotype: A!; isotype: L!)

#### Description.

Trees 15–18 m. Indumentum (branchlets, inflorescence axis, flowers) densely appressed sericeous or sericeous-villous (trichomes yellowish). Branchlets terete to compressed, brown (dried), epidermis smooth, becoming flakey or scaly, sericeous-villous. Leaves opposite, evenly distributed along branchlets, strongly discolorous, internodes 1–3 cm long; venation perfect basal acrodromous, secondary and tertiary veins visible above and below; intramarginal vein closely paralleling leaf margin, 0.5–0.7 mm from margin at midpoint of blade. Colleters absent. Petioles 5–9 mm long, slightly sulcate throughout. Leaf blades 3.8–7.0 cm long, (1.1–)1.5–3.0(–3.5) cm wide, elliptic (rarely broadly elliptic), base cuneate, apex narrowly acuminate, tip acute and somewhat falcate; adaxial surface matte, sericeous but becoming glabrescent, midvein slightly sulcate in proximal half but more or less flush distally; abaxial surface densely sericeous between the secondary and tertiary veins, midvein projecting throughout, oil glands (if present) entirely obscured by indumentum. Inflorescence axillary, flowers solitary (=monads) or in 3-flowered cymes (=botryoids), solitary to paired or fascicled in axils, pedicels of monads up to 1–5 mm long, rigid and ascending. Bracteoles 1.5–3.0 mm long, less than 0.5 mm wide at base, linear, mostly erect or ascending, mostly persistent in flower. Hypanthium 2.5–3.3 mm long, ca. 2.5 mm wide at base of calyx lobes, cupulate, densely hairy. Caly x lobes 2.7–3.0 mm long, broadly obtuse, glabrescent adaxially, densely sericeous abaxially. Petals 5.5–7.0 mm long, 3.0–3.5 mm wide, ovate to narrowly ovate, whitish, mostly glabrous adaxially, densely sericeous abaxially, oil glands common. Stamens 65–75, multiseriate; staminal disk short-hairy; filaments 2–3 mm long; anther sacs 0.3–0.5 mm long, globose to subcylindrical, sub-basifixed or basifixed, crowned by a single large apical gland. Style ca. 4.5 mm long, glabrous; stigma narrow to slightly capitate, prominently papillose. Ovary and locule 1, placentas 2, linear, ovules disposed in regular rows.

**Figure 3.**
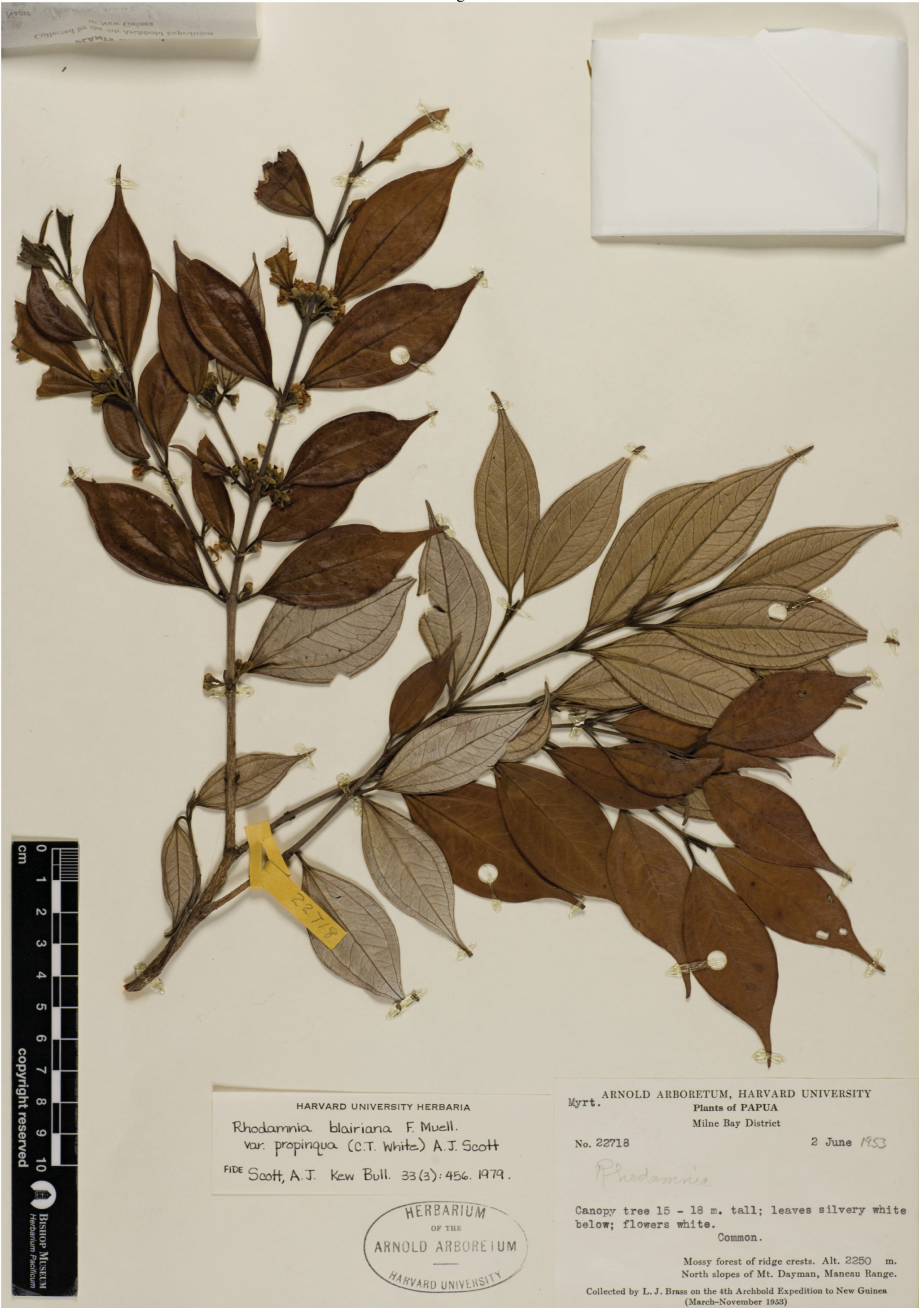
*Rhodamnia daymanensis* N. Snow. Photo of the holotype at A (*L. J. Brass 22718*).

#### Phenology.

Flowering in June; fruiting interval unknown.

#### Distribution.

Papua New Guinea, Milne Bay Province, north slopes of Mt. Dayman in the Maneau Range; mossy forest of ridge crests over metamorphic rocks (see Davies 1980; Daczko et al. 2009) at ca. 2250 meters.

#### Conservation status.

Data Deficient. The collection label indicates the species was common (at least locally) at the time of its collection in 1953. However, the absence of additional collections over the past sixty years suggests that Threatened might more accurately reflect its true status.

#### Comments.

*Rhodamnia daymanensis* appears to be part of the “pearly” group (Snow 2007) by virtue of its abaxial indumentum. Scott (1979) included this specimen in *Rhodamnia blairiana* var. *propinqua* (C.T. White) A.J. Scott.

Among the species of *Rhodamnia* in New Guinea occurring at elevations above 2000 meters with a similar abaxial indumentum, *Rhodamnia daymanensis* mostly closely resembles *Rhodamnia lancifolia*. The type collection of *Rhodamnia lancifolia* (2425 m) is approximately 25 km west of the type locality of *Rhodamnia daymanensis* (2250 m) in similar habitats. However, *Rhodamnia lancifolia* differs by its more narrowly elliptic leaves, a less pronounced petiolar sulcus, slightly impressed adaxial laminar midvein (vs. more deeply impressed in *Rhodamnia daymanensis* in the proximal half), and the more yellowish (and longer, on average) abaxial laminar indumentum. The adaxial leaf surface of *Rhodamnia lancifolia*, for which there are many collections,is nearly black when dried, which contrasts with the fucosus (dark greyish brown, Beentje 2010) dried color of *Rhodamnia daymanensis*. The flowers (presumably hypanthium and abaxial surfaces of calyx lobes and petals) of *Rhodamnia lancifolia* have a brownish-pinkish indumentum (Stevens & Veldkamp LAE 55582; isotype [L!]), whereas the floral indumentum of *Rhodamnia daymanensis* is mostly distinctly yellowish.

### Rhodamnia makumak

N. Snow sp. nov.

urn:lsid:ipni.org:names:77123884-1

http://species-id.net/wiki/Rhodamnia_makumak

Figures 1 4

*Resembling other species of* Rhodamnia *having stellate trichomes but differing by its sessile to subsessile flowers and narrowly elliptic leaves with occasionally falcate apices*.

#### Type.

Papua New Guinea. Milne Bay Province: E. of Mt. Suckling in valley of the upper Maiyu R[iver] c. 15 km WNW of Biniguni airstrip, ca. 9°40'S, 149°10'E, ca. 350 m, 7 Jul 1972, R. Pullen 8433(holotype: A! [no accession numer]; isotypes: BO n.v., BRI!, CANB!, L!, LAE n.v., K!, TNS n.v.).

#### Description.

Trees to 25 m. Buttresses present but low of stature; fluting or twisting absent. Bark of main trunk reticulate-flaky, brownish. Indumentum, where present (branchlets, petioles, abaxial leaf surface, distal portion of adaxial leaf midvein, peduncles, bracteoles, hypanthium, calyx lobes, adaxial petal surfaces), densely tomentose and velvety in texture, consisting of stellate, ferrugineous trichomes. Branchlets terete to compressed. Leaves opposite, more or less evenly distributed along branchlets, discolorous; venation perfect or imperfect suprabasal acrodromous, secondary and tertiary veins faint but visible adaxially, intramarginal vein faintly visible from adaxially, tracing irregularly between tips of secondary veins and ca 0.5 mm from blade margin. Colleters absent. Petioles 4.5–6.5 mm long, rounded in transverse section. Leaf blades 4.5–7.5 cm long, 1.4–2.2 cm wide, narrowly elliptic, base cuneate, margin flat, apex acuminate and sometimes falcate, tip (uppermost 10% of blade) acute; adaxial surface matte, midvein slightly sulcate more or less throughout to sometimes flush distally, tomentose proximally; abaxial surface orangish-velvety by virtue of indumentum, midvein projecting throughout. Inflorescence terminal and lateral, solitary or paired to mostly a fasciculate cluster of monads, the monads sessile or on pedicels up to 3 mm long. Bracteoles 1.8–2.3 mm long, 0.4–0.6 mm wide, linear, rigid, ascending to erect, the apex not reaching base of calyx lobes, persisting. Hypanthium campanulate; anthopodium (if present) up to 1 mm long; metaxyphylls absent. Calyx lobes 4, 2.2–2.7 mm long, 2 (of the 4 lobes) more or less rectangular (length-width ratio 3:2), slightly longer than the 2 shorter, broadly ovate (3:2) lobes; adaxial surface densely tomentose or somewhat less so basally and near margins, abaxial surface densely tomentose throughout. Petals (material sparse) 2–2.5 mm long, 2–2.3 mm wide, elliptic to ovate, tomentose above and below. Stamens ca. 30–40, filaments 2–3 mm long; anther sacs ca. 0.5 mm long, globose, sub-basifixed. Style 3.5–4 mm long, hairy below; stigma narrow to slightly capitate. Fruit not seen.

**Figure 4.**
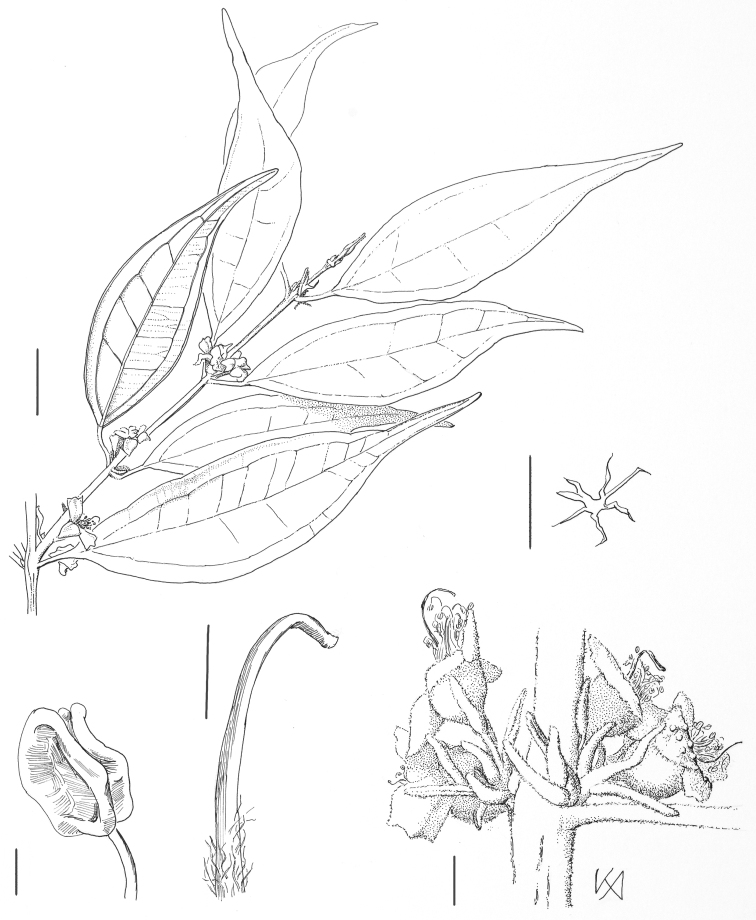
*Rhodamnia makumak* N. Snow. Drawing of isotype at A (*R. Pullen 8433*). Clockwise from top: branchlet (bar = 1 cm); stellate trichome (bar = 0.1 mm); inflorescences (bar = 1 mm); style (bar = 1 mm); dehisced anther (bar = 0.3 mm).

#### Phenology.

Flowering confirmed only for early July but likely also in late June; fruiting unknown but probably June to July and possibly longer.

#### Distribution.

Papua New Guinea, Milne Bay Province; known only from rainforest on a plateau of ca. 350 meters elevation.

#### Conservation status.

Data Deficient.

#### Etymology.

From the local vernacular name “makumak” as a noun in the nominative.

#### Vernacular name.

*Makumak* in the local Daga language.

#### Comments.

*Rhodamnia makumak* ispart of a group of species characterized by a hypothesized synapomorphy of stellate trichomes (Snow 2007). Scott (1979) treated the type collection as *Rhodamnia blairiana* var. *blairiana*, which as presently understood occurs only in Australia (Snow 2007). Dr. Gordon Guymer (BRI), who worked previously on *Rhodamnia*, likewise recognized the affinity of the type collection to *Rhodamnia blairiana*, judging from his specimen annotations.

*Rhodamnia makumak* is said to be a large tree with low buttresses (dimensions of buttresses lacking on label). The best diagnostic characters include the stellate, ferrugineous indumentum on leaves and hypanthium, which imparts a densely velvety appearance; the sessile to subsessile axillary clusters of flowers; and narrowly elliptic leaves bearing an acuminate and sometimes falcate apex. A provisional key to species with stellate trichomes follows.

#### Key to species of *Rhodamnia* with stellate trichomes

**Table:** 

1	Leaves elliptic to broadly elliptic, 11–18 cm long, apex abruptly cuspidate-caudate	*Rhodamnia kamialiensis* N. Snow & W. N. Takeuchi
–	Leaves narrowly elliptic or narrowly ovate to ovate or elliptic, 4–12 cm long, apex acute to acuminate	2
2	Flowers pedicellate, pedicels mostly > 5 mm long; leaf apex acute	*Rhodamnia propinqua* C.T. White
–	Flowers sessile or nearly so; leaf apex acute to acuminate	3
3	Flowers sessile or pedicels to 3 mm; leaf apex acuminate and sometimes falcate	*Rhodamnia makumak*
–	Flowers pedicillate, pedicels 3.5–6 mm long; leaf apex acute to acuminate, rarely mucronate, never falcate	4
4	Seeds with a thin but pronounced equatorial ridge; plants of Australia, 650–1300 m	*Rhodamnia blairiana* F. Muell
–	Seeds lacking an equatorial ridge; plants of Australia and Papua New Guinea, sea level to ca. 500 m	*Rhodamnia sharpeana* N. Snow

### Rhodamnia toratot

N. Snow sp. nov.

urn:lsid:ipni.org:names:77123885-1

http://species-id.net/wiki/Rhodamnia_toratot

Figures 1 5

Superficially resembling species with stellate and typically ferrugineous trichomes but differing by its highly crispate trichomes.

#### Type.

Papua New Guinea. Milne Bay District, Nowata, c. 6 miles W. of Rabaraba, 09°59'S, 149°43'E, ca. 520 m, 5 Jul 1969, R. Pullen 7709 (holotype: K!; isotypes: A! [barcode 00307479], BISH!, BO n.v., BRI!, CANB n.v. [00217141.1 and 00217141.2], G!, L n.v., LAE n.v.).

#### Description.

Trees to ca. 5.5 m. Bark of main bole unknown. Indumentum (branchlets, leaves, flowers, fruit) mostly densely tomentose-lanate, the trichomes highly crisped, ferrugineous, and generally somewhat appressed (see also description of abaxial leaf surface below). Branchlets terete to slightly compressed, reddish-brown (dried); epidermis smooth but finely and evenly striate throughout becoming somewhat fissured with age; oil glands sparse to common (obscured by indumentum on younger branchlets). Leaves opposite, evenly distributed along branchlets, somewhat discolorous; venation perfect basal to slightly suprabasal perfect or imperfect acrodromous, secondary and higher order veins abaxially prominent, the secondaries varying greatly in prominence (and thus hard to estimate numerically) but mostly spaced (2–)3–7 mm along the midvein; intramarginal vein less pronounced than the secondary veins, paralleling leaf margin closely, mostly ca. 0.5–1.0 mm from margin at midpoint of blade. Colleters absent. Petioles 9.0–13.5 mm long, terete, densely lanate-tomentose. Leaf blades 6.5–12.0 cm long, 3.1–5.2 cm wide, elliptic to ovate, base cuneate to nearly rounded, apex acute to acuminate, tip acute to acuminate; adaxial surface matte, initially lanate but becoming glabrescent, midvein slightly impressed throughout; abaxial surface lanate along midvein and secondary veins when younger, increasingly glabrous with age, densely/minutely hoary between veins, midvein raised prominently throughout. Inflorescence lateral in current season’s growth, flowers solitary to mostly densely fascicled, sessile to subsessile with pedicels up to 5 mm long, the pedicels lax, sometimes bending. Bracteoles ca. 2.0–2.5 mm long, ca. 0.5 mm wide at base, linear, persisting in flower and frequently in fruit. Hypanthium cupulate. Calyx lobes 2.5–3.5 mm long, ovate, apex obtuse, densely hairy abaxially but adaxially less so (especially proximally) with age, persisent and erect in fruit. Petals (material scanty), ca. 4.0–4.5 mm long, up to 3.5 mm wide, broadly obovate, more or less glabrous adaxially, densely lanate abaxially. Staminal disk ca. 4.5 mm in diameter; staminal ring narrow, shortly villous-lanate (trichomes whitish-yellow). Stamens numerous (estimated 75–105); anthers sacs (material scanty) cylindrical, ca. 1.0 mm long, bearing a single apical gland; filament length unknown. Styles not seen, but persisting bases densely lanate in fruit. Ovary with 1 locule; placentas 2; placentation parietal; ovules numerous. Fruit subglobose, 7.5–8.5 mm long (probably immature) x 8.0–9.5 mm wide, greenish when young but becoming brownish on account of dense indumentum. Seeds somewhat compressed, ca. 1–2 mm thick.

**Figure 5.**
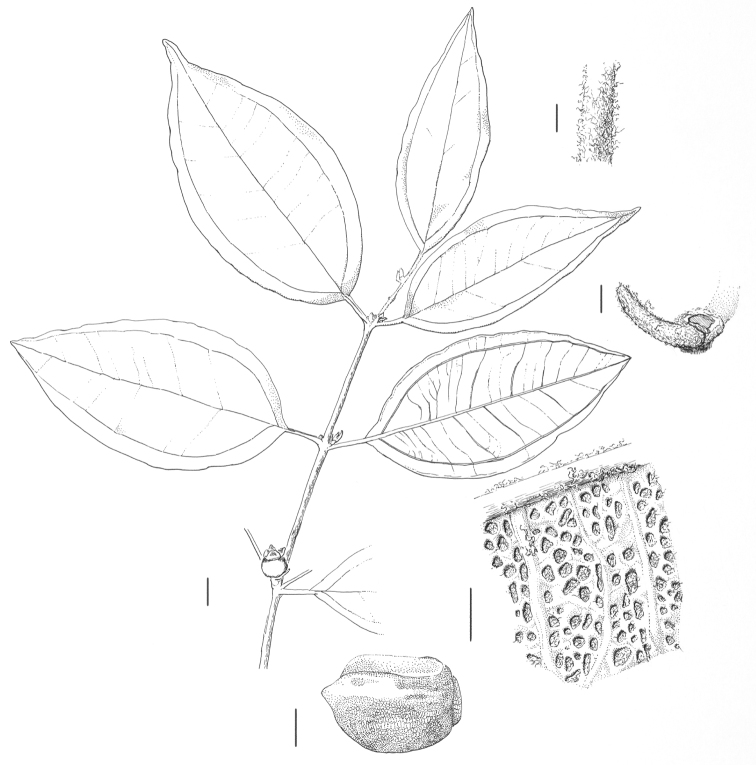
*Rhodamnia toratot* N. Snow. Drawing of isotype at BISH (*R. Pullen 7709*). Clockwise from top center: branchlet; detail of branchlet indumentum; bracteole; abaxial leaf surface; seed. Scale bars = 1 mm, except branchlet bar = 1 cm.

#### Phenology.

Flowering unknown; fruiting in July.

#### Distribution.

Milne Bay Province in Papua New Guinea; in secondary (regrowth) forest with *Dodonaea* Adans. (Sapindaceae) and *Castanopsis* (D. Don) Spach (Fagaceae).

#### Conservation status.

Data Deficient; possibly Threatened for same reasons cited above for *Rhodamnia asekiensis*.

#### Etymology.

The specific epithet is derived from *toratot* as a noun in the nominative.

#### Vernancular name.

Locally known as *toratot* in the Nowata language.

#### Comments.

The tomentose to lanate indumentum on the branchlets and inflorescences (Fig. 5) of *Rhodamnia toratot* suggests its inclusion in the “villous” group of species (Snow 2007), but the minute trichomes between the tertiary and higher-order venation on the abaxial leaf surface suggest possible inclusion in the “hoary” group (Snow 2007).

Scott (1979) had assigned the type gathering of *Rhodamnia toratot* to *Rhodamnia blairiana* var. *propinqua*.However, the two taxa are easily distinguished based on trichome density and appearance. The abaxial laminar indumentum of *Rhodamnia toratot* is characterized by the highly contorted form of the individual trichomes, which are more sparsely distributed than the densely stellate trichomes present on the type specimen of of *Rhodamnia blairiana* var. *propinqua*.

### Rhodamnia waigeoensis

N. Snow sp. nov.

urn:lsid:ipni.org:names:77123886-1

http://species-id.net/wiki/Rhodamnia_waigeoensis

Figures 1 6

*Closely resembling but differing from* Rhodamnia novoguineensis *by its thicker and more rigid pedicels, thickly coriaceous leaves, basal acrodromous venation, densely yellowish abaxial laminar indumentum, and solitary flowers*.

#### Type.

Indonesia. Waigeo Island, Go Isthmus, path from Poean Bay to Fofak Bay, 17 Feb 1955, P. van Royen 5556(holotype: A! [bar code no. 00307477]; isotypes: CANB!, K!, L n.v.).

#### Description.

Trees 5–7 m; girth to 15 cm. Branchlets terete to slightly compressed, the epidermis later becoming fissured; indumentum densely sericeous, mostly yellowish or somewhat ferrugineous but becoming more whitish with age. Leaves opposite, evenly distributed along branchlets, discolorous, glossy above and below, the nacreous sheen below imparted by the dense, tightly appressed greenish-white indumentum. Colleters absent. Petioles 5–6.5 mm long, somewhat flattened above, densely sericeous (or somewhat tomentose with age), the indumentum yellowish but aging whitish. Leaf blades (3.5–)6.0–10.0 cm long, (1.8–)2.5–3.7 cm wide, narrowly ovate to ovate, surface flat or slightly wavy; base cuneate, apex acuminate and occasionally somewhat falcate, tip acute; venation perfect basal acrodromous; secondary veins numerous but thin, ca. 0.8–2.0 mm apart; marginal nerve prominent, mostly 0.7–0.9 mm from mid-leaf margins; margins flat; adaxial surface sparsely sericeous, midvein flush throughout, oil glands invisible; abaxial surface densely sericeous with greenish-whitish indumentum but this mostly not obscuring venation, midvein raised throughout, oil glands invisible. Inflorescence (limited material) a 3-flowered cyme, terminal, solitary (one per leaf subtending leaf); peduncle ca. 5 mm, stiff, terete in transsection, densely yellowish-orangish sericeous; pedicel to ca. 3 mm long, indumentum as per peduncle. Bracteoles 2, narrowly triangular and stiffly erect, ca. 2 mm long, ca. 0.5 mm wide, sericeous, sometimes persisting into fruit. Hypanthium cupulate, densely sericeous, oil glands absent, texture smooth. Calyx lobes 4, 2.3–3.5 mm long, broadly ovate, sericeous above, densely sericeous below, more or less reflexed in fruit. Petals 4, 5.5–7.0 mm long, width uncertain (material scanty), apparently obovate to broadly obovate, white (based on specimen label), sparsely sericeous above, densely sericeous below. Staminal disk 2.5–3.5 mm wide, densely short-hairy. Ovary apex densely short-hairy. Stamen number uncertain but almost certainly greater than 20, filaments and anthers red (from specimen label); anthers subcylindrical (material scanty), ca. 0.5 mm. Stigma not seen. Locule 1, placentation parietal, placentas 2, ovules numerous. Fruit (reportedly immature) globose-subglobse, up to 8 mm long and 9.5 mm wide, light green when immature, densely sericeous but indumentum thinning with maturity. Seeds irregularly angular, up to 4 mm long (small sample), up to 9 per fruit, crowded; seed coat hard. Embryos not seen.

**Figure 6.**
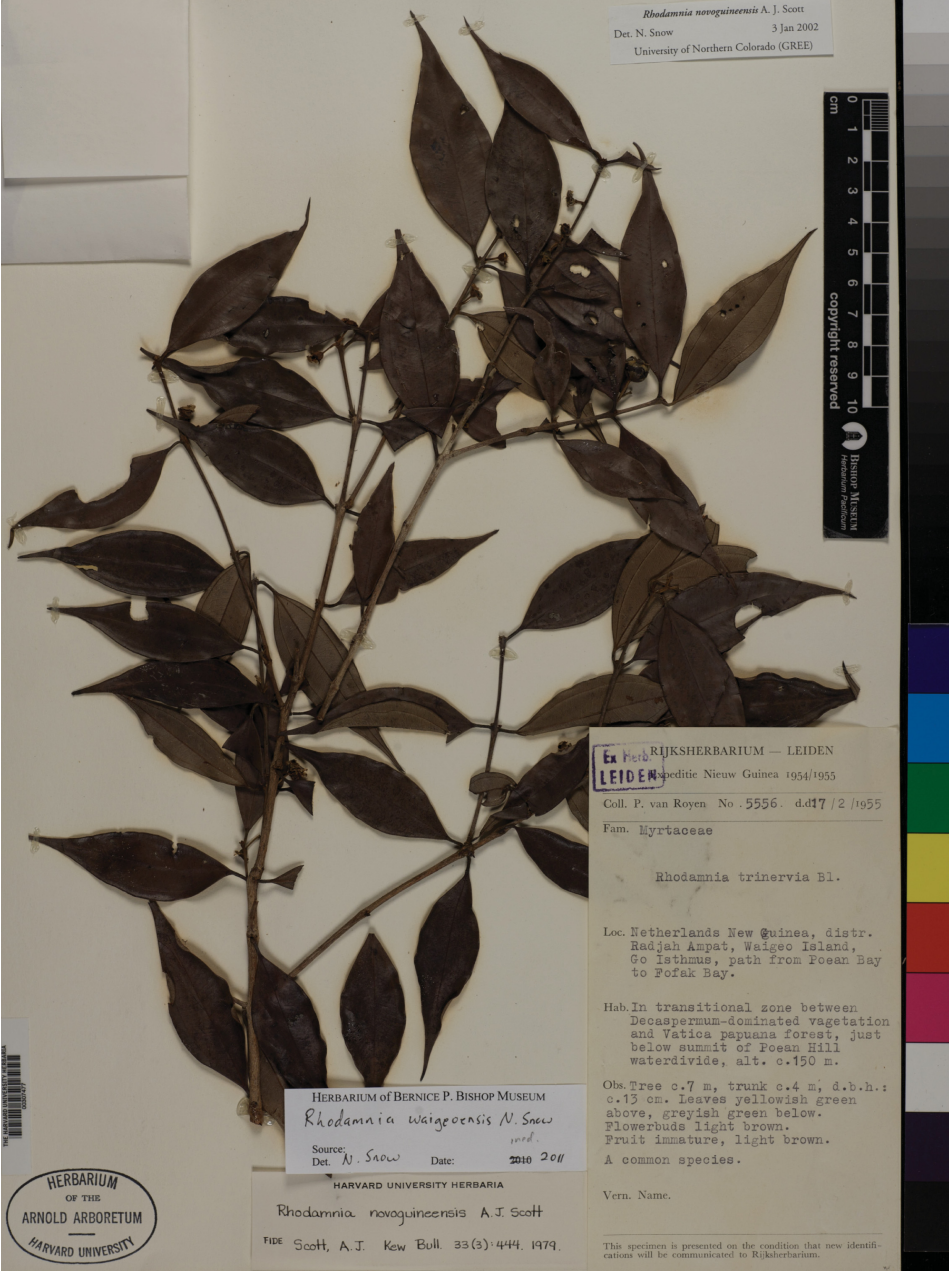
*Rhodamnia waigeoensis* N. Snow. Photo of the the holotype at A (*P. van Royen 5556*)

#### Phenology.

Flowering February; fruiting in January and February.

#### Distribution.

Waigeo Island, Indonesia; from ca. 10–150 m elevation in xerophytic, Myrtaceae-dominated vegetation at lower elevations behind and upslope of the village of Waifoi, and from transitional forests dominated by *Decaspermum* J.R. Forst. & G. Forst. (Myrtaceae) or *Vatica rassak* Blume(=*Vatica papuana* Schum. & Hollr. [synonym]) (Dipterocarpaceae) at the higher elevation (ca. 150 m).

#### Conservation status.

Data Deficient given the lack of recent information or collections. *Rhodamnia waigeoensis* is presently known only from two collections. The specimen on the type label indicates that the species was common locally at the time of its collection nearly sixty years ago. A vegetation type similar to that of the type gathering occurs on the island of Rauki, where the species also may occur. While the reported ethnobotanical use of *Rhodamnia waigeoensis* for cigarette making may lend the species some protection, it also may have encouraged overexploitation.

#### Vernacular name.

*Kikir* (in the Malayan language).

#### Ethnobotany.

The herbarium label indicates that the leaves are used for making cigarettes.

#### Comments.

*Rhodamnia waigeoensis* belongs in the “pearly”group of species given its nacreous indumentum (Snow 2007). Scott (1979) included the type gathering of *Rhodamnia waigeoensis* in *Rhodamnia novoguineensis* A.J. Scott and the paratype gathering in *Rhodamnia pachyloba* A.J. Scott.

*Rhodamnia waigeoensis* differs from *Rhodamnia novoguineensis* by its thickly coriaceous leaves (vs. thinly coriaceous in *Rhodamnia novoguineensis*), consistently basal acrodromous leaf venation (vs. even or uneven suprabasal acrodromous in *Rhodamnia novoguineensis*), dense abaxial laminar indumentum with yellowish trichomes (vs. relatively sparse and whitish in *Rhodamnia novoguineensis*), solitary flowers (vs. triads or few-flowered racemes in *Rhodamnia novoguineensis*), thicker (0.5–0.7 mm wide) and rigid pedicels (vs. ca. 0.3 mm thick and flaccid in *Rhodamnia novoguineensis*).

Waigeo Island is part of the Raja Ampat Islands of Indonesian New Guinea. The region harbors unusual vegetation assemblages (van Royen 1960), has high rates of endemism (Supriatna 1999), and was the subject of relatively recent rapid-assessment surveys (Takeuchi 2003a). Van Royen (1960: 54–56) summarized the vegetation on portions of Waigeo Island using six broad categories. One of these, xerophytic vegetation, is described as having three variants, one being dominated by Myrtaceae.

The label of the type specimen refers directly to the xerophytic vegetation located behind the small village of Waifoi on the east bank of Majalibit Bay. Takeuchi (2003a,b) reported that the Waigeo ultrabasic vegetation resembles the pioneer communities on the ultrabasics at the Kamilai Wildlife Management Area (KWMA) in the Bowutu Mountains (Morobe Province, Papua New Gueina). Communities at KWMA can be topographically unstable due to landslides, but in general appearance and composition are similar to those on Waigeo. However, Takeuchi (2003b) believes the vegetation on the Waigeo ultrabasics is primarily caused by fire succession.

A xerophytic vegetation similar to that occuring on the hills upslope of Waifoi, the village near the type collection, was encountered elsewhere by van Royen (1960: 39, 41) in the Kambelay Hills and the Go Isthmus of Waigeo Island. This general type of xerophytic vegetation is said to recur on Rauki Island, which lies northwest of Kabaré Bay, where it occurs at the higher elevations (probably less than ca. 40 m, but reported by van Royen [p. 45] as 25 m) along the southern end of the island (van Royen 1960: 44) at ca. 0°52'S, 130°56'E (coordinates based on Google Earth™ [accessed 2 June 2009]). (Rauki Island has been known previously as Rawak, Rawah or Lawak [van Royen 1960: 43]). The substrates underlying the xerophytic vegetation of Rauki include ultrabasic outcrops among the more prevalent limestone (van Royen 1960: 45).

Van Royen (1960: 32) described the soils underlying the xerophytic vegetation on Waigeo as “sandy brown clays with much limestone”. The relatively open vegetation on the slopes was indicated as being spare of trees but conspicuous in its presence of shrubby Myrtaceae. Noted specifically for Myrtaceae (van Royen 1960: 32, 55, 59) were *Baeckia frutescens* L., *Myrtella beccarii* F. Muell. and *Decaspermum rubrum* (Blume) Baill. (as *Decaspermum fruticosum* J. R. Forst. & G. Forst.var. *rubrum*, a nomenclatural change that was apparently never validly published [Scott 1985; Govaerts et al. 2008]), and “*Rhodamnia trinervia* Reinw. ex Blume*”*. However, the collection number (5556) that van Royen (1960: 59) cited for *Rhodamnia trinervia* represents the holotype of *Rhodamnia waigeoensis*, and *Rhodamnia trinervia* is now considered to be a synonym of *Rhodamnia rubescens* (Benth.) Miq. (e.g., TPL 2012).

#### Specimen examined.

West Papua (Papua Barat; as Radjah Ampat on label), Waigeo Island, Waifoi on E bank of Majalibit [= Mayalibit] Bay, 18 Jan 1955, P. van Royen 5227 (L).

### Updated distribution for *Rhodamnia sharpeana*

Snow (2007) previously described *Rhodamnia sharpeana* from Queensland, Australia, where it occurs in rainforests between 50–500 m, ranging from the vicinity of Isabella Falls north throughout much of Cape York Peninsula. The specimen cited below from Kamiali Wildlife Management Area (KWMA) in Papua New Guinea extends the range north by ca. 650 km from the collections on Cape York Peninsula, whereas the specimen from Tagala (Tagula) Island extends its range eastwards approximately 1000 km from the collections in North Kennedy District, Queensland (Snow 2007). The specimen from Kamiali also increases the known altitudinal range by ca. 160 meters and represents a first report of the species occurring over ultrabasic substrates (Takeuchi 2003; Snow and Takeuchi 2009).

**Phenology.** Flowering September through December; fruiting presumably October through at least February (Snow 2007).

**Distribution.** Northeastern Australia to east-central and southeastern Papua New Guinea;rainforests (including ultrabasics at Kamiali in Papua New Guinea) to margins of anthropogenically derived grassland (*Brass 28176*); near sea level to 500 m.

**Conservation status.** Least Concern (IUCN, 2010) based on its known distribution and abundance.

**Comments.** The known range of *Rhodamnia sharpeana* is now considerably wider than previously understood (Snow 2007). In Papua New Guinea it occurs on floodplains and along streambanks in Kamiali Wildlife Management Area (KWMA), and in anthropogenically derived grasslands on Tagula Island in the Louisiade Archipelago (Fig. 1). Takeuchi (2003b) suggested that the effect of ultramafic substrates on plant distributions is reduced with increasing levels of humidity. The presence of *Rhodamnia sharpeana* in high rainfall environments at KWMA may not be surprising, given our newer understanding of its wider geographical distribution.

**Specimens examined.** Papua New Guinea. Morobe Province, Kamiali Wildlife Management Area; alluvial floodplain along Saia (Sela) River, 7°21.5'S, 147°08'E, ca. 25 m, 25 May 2000, W. Takeuchi 14405(K!); near mouth of Saia River at mount of Hessen Bay, alluvial flatland forest, sea level, 7°21.7'S, 147°08.3'E, common riverbank shrub, 4–5 m height but forest occurrences much taller; leaves dry-textured, adaxially dark dull green, petals white, W. Takeuchi & A. Towati14841(K!); ridge to Blue Mt, 7°17'29"S, 147°05'12"E, ca. 762 m, 28 Feb 2005, W. Takeuchi & D. Ama 18977(BISH (736568! and 736569!), LAE [n.v.]). Milne Bay Province, Sudest [= Tagula or Tagala] Island, (inland from) Rambuso, 300 m, 20 Sep 1956, L. J. Brass 28176(BISH 733105!; A n.v., K n.v., L n.v., US n.v.).

## Discussion

Sixteen new species of *Rhodamnia*, including the five newly proposed here, have been described since Scott’s (1979) generic revision (Guymer and Jessup 1986; Guymer 1998; Snow and Guymer 1999; Snow et al. 2001; Snow 2007; Snow and Takeuchi 2009). Apart from the new species and those that Scott (1979 and see above) treated in other taxa, the species of *Rhodamnia* that I recognize for New Guinea differ from Scott (1979) in only two ways. First, I follow White (1951) and recognize *Rhodamnia propinqua* as distinct from the Australian *Rhodamnia blairiana*. Second, I maintained the Australian species *Rhodamnia spongiosa* (F.M. Bailey) Domin as distinct from the New Guinea species *Rhodamnia glauca* Blume (Snow 2007).

Additional studies still are clearly needed in *Rhodamnia*. The phylogeny of the genus has never been established, and the hypothesized informal groups of *Rhodamnia* (Snow 2007) need testing. All new collections from New Guinea and Malesia deserve close scrutiny. Scott (1979) adopted a broad taxonomic concept of *Rhodamnia cinerea* Jack and probably was correct to synonymize many names therein given its wide geographical distribution. However, I have seen newer collections from Malesia that may merit taxonomic recognition, so a thorough review of the Malesian species, including *Rhodamnia cinerea*, is now warranted given the many recent collections across that region.

Efforts also should be made to recollect all species of *Rhodamnia* in New Guinea given the paucity of specimens for many species and the present inability to assign conservation threat assessments with high levels of confidence. Two suggestions for collecting in remote, biodiverse areas are worthy of repeating. First, as Takeuchi (2000) expressed for New Guinea generally, survey botanists should collect uncritically while doing inventories, since taxonomic novelties and range extensions often are discovered only many years later in the herbarium by taxonomic specialists (Bebber et al. 2010; Snow et al. 2012), and because specimens that appear to be known species may in fact be taxonomic or geographical novelties. Second, workers are encouraged to include observations of local relative abundance for each specimen, given the value that such information can provide for later conservation threat assessments (Snow 2011: 687-688), however tentative they may be.

## Supplementary Material

XML Treatment for Rhodamnia asekiensis

XML Treatment for Rhodamnia daymanensis

XML Treatment for Rhodamnia makumak

XML Treatment for Rhodamnia toratot

XML Treatment for Rhodamnia waigeoensis
